# Top 10 health care ethics challenges facing the public: views of Toronto bioethicists

**DOI:** 10.1186/1472-6939-6-5

**Published:** 2005-06-26

**Authors:** Jonathan M Breslin, Susan K MacRae, Jennifer Bell, Peter A Singer, the University of Toronto Joint Centre for Bioethics Clinical Ethics Group

**Affiliations:** 1 University of Toronto Joint Centre for Bioethics, 88 College Street, Toronto, Ontario, M5G 1L4, Canada; 2 Department of Medicine, University of Toronto. Joint Centre for Bioethics, 88 College Street, Toronto, Ontario, M5G 1L4, Canada

## Abstract

**Background:**

There are numerous ethical challenges that can impact patients and families in the health care setting. This paper reports on the results of a study conducted with a panel of clinical bioethicists in Toronto, Ontario, Canada, the purpose of which was to identify the top ethical challenges facing patients and their families in health care. A modified Delphi study was conducted with twelve clinical bioethicist members of the Clinical Ethics Group of the University of Toronto Joint Centre for Bioethics. The panel was asked the question, what do you think are the top ten ethical challenges that Canadians may face in health care? The panel was asked to rank the top ten ethical challenges throughout the Delphi process and consensus was reached after three rounds.

**Discussion:**

The top challenge ranked by the group was disagreement between patients/families and health care professionals about treatment decisions. The second highest ranked challenge was waiting lists. The third ranked challenge was access to needed resources for the aged, chronically ill, and mentally ill.

**Summary:**

Although many of the challenges listed by the panel have received significant public attention, there has been very little attention paid to the top ranked challenge. We propose several steps that can be taken to help address this key challenge.

## Background

It is not uncommon for health care professionals to clash with the family of the patients for whom they care over treatment decisions. Some patients will inevitably suffer the consequences of an error made during their care or hospitalization. Many people in need of diagnostic tests or surgical procedures are forced to wait months, and perhaps even years, to receive these services. These are just some examples of the kinds of ethical challenges that patients and their families may confront in the health care setting.

Although these challenges have been discussed widely in the literature as isolated ethical issues in health care, no attempt has ever been made to collate and prioritize them. Ranking the top ethical challenges facing the public can be an effective and valuable way of bringing them to the public's attention. Moreover, efforts to address ethical challenges in health care vary significantly from one to another, with some receiving a great deal of attention from the media and from government, while others go largely unnoticed; it would be valuable to discover whether the attention given to these challenges is allocated appropriately. Therefore, the purpose of this study was to identify the top ethical challenges facing patients and families in health care, from the perspective of a panel of clinical bioethicsts.

### How the study was conducted

A modified Delphi study was conducted with twelve clinical bioethicist members of the Clinical Ethics Group of the University of Toronto Joint Centre for Bioethics. The justification for using a panel of bioethicists rather than a panel of community members is that clinical bioethicists will have a greater familiarity with the overall range of challenges than community members due to the fact that the ethical challenges are highly concentrated in their day-to-day work.

These clinical bioethicists work in a wide range of health care institutions, including quaternary-level institutions (for both adult and pediatric care), geriatrics/long-term care, rehabilitation, addiction and mental health, and community hospitals. In addition, the experience of the panel members covers both inpatient and outpatient health care. The Clinical Ethics Group at the Joint Centre for Bioethics is the largest institutionally-affiliated collection of clinical bioethicists in Canada, and perhaps in the world. Most of the panel members have several years of experience in clinical ethics, and the Clinical Ethics Group as a whole conduct more than 1200 consults per year. We believe that due to their extensive experience in ethics consultation and bioethics research, this group would be able to offer a uniquely informed perspective on the ethical challenges facing patients and their families. The twelve panel members chosen for the study represent a subset of the larger Clinical Ethics Group; although other members of the Clinical Ethics Group participated in various steps of the process, only the results of the twelve panel members who participated in all rounds were included in the results.

In the first round of the process the bioethicists were provided with a list of 38 themes that summarized the themes discussed during the previous two years of case conference discussions at the Joint Centre for Bioethics. The bioethicists were asked to provide a list of what they believed to be the top ten ethical challenges facing the public, which they could pull from the list of 38 themes or provide additional themes in their own words. In this context the phrase "ethical challenges facing the public" was meant to imply issues, situations, or problems, which have ethical implications, and would impact or affect the public either directly or indirectly. Although there were no formal criteria for determining the relative impact of the various challenges, the panel members considered such factors as the prevalence of the challenge (how often it occurs and is likely to occur in the future), how many patients and families are and will be affected by the challenge, and the seriousness of the impact on the public.

The panel members each responded by email with their list of ranked challenges, along with brief explanations as to why their chosen items were important and challenging. Two of the authors (SKM and JB) then clustered and reworded the themes as necessary to meet a desired level of specificity, and any themes from the original list not ranked by any panel member were dropped from the list. Following this, a list of 32 items was fed back to the panel in advance of a face-to-face meeting. The main purpose of this meeting was for the group to narrow the list further by grouping similar challenges together to make sure that all items were in fact distinct challenges. From this meeting a new list of 23 items was circulated for the second round of ranking, in which participants were again asked to rank their top ten items and give rationales for their rankings. This third round of ranking produced the final list of top ten challenges. The process was stopped after the third round because the list of challenges could not be specified or differentiated any further. The overall ranking was done as per the Delphi method, whereby the highest ranked scenario cited by a panel member was then assigned ten points, followed by the second highest ranked scenario receiving nine points, and so on until the tenth highest ranked scenario. Individual scores were summed up to create a total score for each scenario and a corresponding ranked list for the group. The maximum score that could be achieved by a single item was 120, which would result only if the same item was ranked as the top challenge by all twelve panel members.

## Discussion

Results of the study are listed in Table 1. In addition to the rankings, the comments from the panel members made during both the face-to-face meeting and the third round of ranking were collected and serve as the basis for much of the content in the Discussion section. With a total of 113 out of a possible 120 points, the highest ranked ethical challenges facing the public in health care was disagreement between patients/families and health care providers over treatment decisions. According to the panel, these disagreements typically take one of two forms: health care professionals might push a treatment option (either for more or less treatment) that patients and families deep unacceptable, or conversely patients/families may push a treatment option (more or less treatment, or different treatment as in alternative or complementary treatments) that health care professionals deem unacceptable. We expand on this challenge in the "Discussion" section of the paper below.

**Table 1:** Top 10 ethical challenges facing Canadians in health care

**Rank**	**Scenario**	**Score**
1	Disagreement between patients/families and health care professionals about treatment decisions	113
2	Waiting lists	102
3	Access to needed health care resources for the aged, chronically ill and mentally ill	89
4	Shortage of family physicians or primary care teams in both rural and urban settings	82
5	Medical error	76
6	Withholding/withdrawing life sustaining treatment in the context of terminal or serious illness	56
7	Achieving informed consent	43
8	Ethical issues related to subject participation in research	40
9	Substitute decision-making	38
10	The ethics of surgical innovation and incorporating new technologies for patient care	21

The second highest ranked ethical challenge facing the public in health care, with 102 total points, was waiting lists. This has been a growing problem in Canadian health care as progressively increasing demand for health care services has put mounting pressure on the already strained Provincial health care systems in the country. According to the panel, waiting for needed care may in some cases compromise the health status and outcomes of patients, impede their ability to return to normal functioning at work and at home, and may also contribute to psychological distress. Waiting lists may also contribute to inappropriate use of scarce resources as is the case when acute care beds are used for long-term care patients, or ICU beds for chronic care patients. Waiting lists also raise the issue of geographical inequities among regions or various health centres.

The third highest ranked challenge was issues related to access to needed health care services for the aged, chronically ill and mentally ill. There are two components to this set of issues: one, according to the panel, is the marginalization of populations such as the elderly and mentally ill due to negative attitudes of many citizens toward those populations. The other component is the historical lack of priority of the needs of these populations in the funding allocation schemes of Canadian health care: the bulk of the funding has traditionally gone toward acute, life-saving care, while long-term care, rehabilitation care, and mental health have been grossly under-funded. According to the panel, socially or economically disadvantaged or mentally ill patients require appropriate advocacy to ensure their needs are met. Lack of patient compliance or self-care is sometimes used as reasons to withdraw resources. According to the panel members, we have an ethical obligation to acknowledge and challenge discriminatory beliefs around age, culture, and mental illness that are culturally and socially constructed in order to reduce the risk of emotional and physical harms of the vulnerable in our hospitals and nursing homes. Often these issues emerge when resources are limited.

The fourth ranked challenge was the shortage of family physicians or primary care teams in both rural and urban settings. According to a 2002 study published by the Canadian Institute for Health Information, the proportion of Canadian medical graduates starting practice as a general or family practitioner dropped sharply, from a high of 80% in 1992 to only 45% in 2000 [1]. This has become such a significant problem in Canadian health care that it was one of the major issues in the recent contract negotiations between the Ontario government and the Ontario Medical Association [2,3]. Many Canadians living in rural areas simply do not have family physicians; in urban settings many patients have to wait so long to see their family physicians that some choose to seek care in emergency rooms as an alternative. This just puts added pressure on already stressed emergency rooms in major Canadian cities. The shortage of family physicians is of considerable concern for a country whose health care system is centred on universal and reasonable access to medically necessary health care services.

The fifth ranked ethical challenge facing the public by the panel was the issue of medical error. Although errors have always been part of medicine, it wasn't until the 1999 report from the Institute of Medicine in the U.S., *To Err is Human: Building a Safer Health System *[4], that the public was made aware of how common medical errors actually are. Examples of such errors can include things that affect single patients, such as a patient receiving the wrong prescription or dosage of medication, or a patient having the wrong surgery performed, or things that impact a larger patient group, as when a hospital fails to properly sterilize surgical equipment. Although medical errors do not in themselves represent an ethical challenge per se, they do carry with them significant ethical implications. For instance, the prevalence of medical errors raises such ethical questions as if, under what circumstances, and how medical errors should be disclosed to patients and/or families.

Sixth on the list, but well behind the issue of medical error in overall scoring, was the challenge associated the appropriate use of pain medication in the terminally or chronically ill, and the use of palliative care at the end of life. For example, health care providers sometimes struggle with how to use pain medication appropriately for terminally ill patients because treating the patient's pain sufficiently can potentially hasten the death of the patient. The panel has suggested that this is one of the contributing factors behind the widespread under-treatment of pain in the terminally and chronically ill. Another challenge that falls into this category surrounds the timing of palliative care, i.e., decision making around when is the appropriate time to shift from a curative to a palliative approach to care.

Seventh on the list according to the panel was the challenge of obtaining informed consent in the health care setting. Research [5] and experience of the panel have shown that there is a huge gap between informed consent in theory and informed consent in practice: many patients do not or *cannot *read the consent forms they're asked to sign; consent discussions and capacity assessments are often superficial and rushed due to time constraints; and those same time constraints often contribute to staff not using interpreters with patients whose first language is other than English. The implication of this is that many patients may be subjected to medical interventions without providing properly informed consent. Since the ethical principle of respect for patient autonomy, on which the doctrine of informed consent is based, has become a central and foundational principle in modern Western health care, the implication of this challenge is troubling.

The eighth top challenge was a family of issues associated with participant involvement in research. There are a wide range of ethical issues related to research in the health care setting, including obtaining informed consent, the balance between providing participants with fair compensation and the risk that the compensation will be a coercive influence, the challenge of balancing benefits and risks of the research, issues around patient privacy and confidentiality, and the ethical appropriateness of involving in research participants who are not capable of giving an informed consent.

The ninth ranked challenge, finishing closely behind the challenges associated with research, was the challenge of substitute decision making. When a patient is incapable of making a particular health care decision, the health care team will turn to the substitute decision maker to make the decision. Depending on the particular jurisdiction there may be a legal hierarchy of decision makers, which typically places the patient's most intimate relationship at the top (spouse or partner) and other relatives toward the bottom of the hierarchy (many Canadian provinces and US states have such a hierarchy written into health care consent legislation). In the experience of the panel members, substitute decision makers often find this task to be a heavy burden, and struggle with the responsibility attached to making a potentially life-altering (and often life-ending) decision on behalf of their loved ones. This burden is experienced to the greatest degree when no guidance has been provided by the patient as to what his or her wishes would be in the current circumstances. When there is no guidance from the patient, conflict often ensues between the health care providers and the family/substitute decision makers as to what would be in the patient's best interests.

Finally, the tenth ranked challenge was that of surgical innovation. This is a challenge that patients and families will only face indirectly, as the general public is likely unaware of what the issues are related to surgical innovation. Surgical innovation raises such questions as, should innovative surgical techniques be considered research and be required to go through research ethics approval? Since variation is often part of the routine process of perfecting surgical techniques, it becomes difficult to ascertain when a surgical innovation becomes an experiment that requires research ethics approval. Also, what protections should be in place to ensure that innovative techniques or procedures can be developed while the risks to patients are minimized?

There are a number of benefits that can be realized with an exercise focused on ranking the top ten ethical issues the public may face. These include providing new contributions to knowledge, raising public awareness, and re-focusing attention on the top challenge. These benefits will be discussed in the discussion section below.

### Providing new contributions to knowledge

The issues described as top ethical challenges by the panel have all been discussed individually in the literature, some extensively. And there have been a few attempts in the past to elicit the views of particular groups on major ethical issues in specific areas. For example, Ersek at al. surveyed a group of oncology nurses to elicit the ethical issues determined to be most important to that group [6]. Along slightly different lines, Walker et al. interviewed a group of physicians and nurses to elicit their perception of "ethics problems" in the care of their patients [7]. However, these previous studies have typically focused on the views of a specific group of health care professionals on ethical issues in particular health care contexts. No attempt has ever been made to seek the opinion of clinical bioethicsts who are in a unique position to offer comment on the overall ethical issues in the health care system. Furthermore, despite extensive coverage of ethical issues in the healthcare literature, no systematic effort has been made to collate and rank these kinds of issues from the perspective of the impact on the public.

### Raising public awareness

A second benefit of such an exercise is that it can be part of an effective strategy to bring these challenges to the public's attention. Another component of the public awareness strategy might include a press release or other form of media attention coordinated with the publication of the research paper. Even the paper itself can spark discussion and bring the issues to the public's attention. Not only would this help to inform the public about ethical challenges they may confront in the health care system to they can be better prepared for those challenges, but it can help garner the public's support in advocating for steps to be taken to address the top challenges.

The challenges described by the panel will impact patients and their families in different ways and to varying degrees. For example, waiting lists (ranked 2^nd^) and the shortage of family physicians (ranked 4^th^) are challenges that will likely have an impact that is felt directly by a large percentage of the public. The same can be said of the third ranked challenge, access to needed health care resources for the aged, chronically ill, and mentally ill, and that challenge will impact an increasing number of patients and families in the future as our populations age and the number of elderly and chronically ill patients rise. This direct impact on the public, combined with the attention that issues like waiting lists do receive in the media, means that some of the challenges described by the panel are already at the forefront of the public's attention. On the other hand, the public is likely to be largely unaware of some of the other challenges mentioned by the panel. These are the challenges that tend to impact a smaller number of patients and families, such as issues related to participation in research (ranked 8^th^), or may impact patients and families more indirectly, such as the issues related to surgical innovation (ranked 10^th^).

### Re-focusing attention on the top challenge

The most interesting result of this study is that the ethical challenge ranked highest by the panel is a challenge that actually receives very little attention in the popular media and at the level of government, and a challenge of which most members of the public are likely completely unaware. It is not surprising, however, that a panel of clinical bioethicists ranked disagreements between patients/families and health care professionals over treatment decisions as the top ethical challenge facing the public in health care. It is not surprising because it is probably the most common reason for requests for ethics consultations, and an area in which many bioethicists focus their research activities. A 2001 study by DuVal et al. found that the most common trigger for ethics consultations among U.S. internists was a desire for help to resolve a conflict [8]. Although the most common arena in which these disagreements occur is the intensive care unit, they can and do occur between patients/families and health care professionals in virtually every health care context: palliative care, rehabilitation, mental health, surgery, general internal medicine, family medicine, and so on. These conflicts can be as serious as an emotionally charged fight over a decision to withdraw aggressive treatment from a terminally ill patient in the intensive care unit, or as mundane as a family physician refusing to acquiesce to a patient's request for antibiotics for a viral infection.

According to the panel, it's the end-of-life critical care cases that tend to be the most emotionally charged, and the most intractable, because these are the cases in which the most is at stake – they typically amount, literally, to conflicts over life and death. A paradigm example of what has become the most common scenario would involve a patient in the late stages of a terminal illness, such as cancer with multiple metastases, or an elderly patient with multiple co-morbidities, who is ventilated in the intensive care unit. The family would be demanding that "everything" be done to maintain the patient's life, while the team feel strongly that subjecting the patient to aggressive interventions would amount to torture. Emotions run high, conflict ensues, and communication inevitably breaks down.

The above is a paradigm example of what is often referred to in the literature and by health care professionals in the clinical setting as a "futility" case. Although there are volumes of literature on the problems associated with the definition and use of the concept of futility, health care professionals know exactly what is meant when a colleague uses the concept: the likely harms of the aggressive intervention(s) outweigh the potential benefits to such a degree that subjecting the patient to the intervention(s) violates their professional (and sometimes personal) values. From the perspective of the health care professionals, the "right" decision is obvious and they cannot understand why the family doesn't see it the way they do. This often leads to these families being labelled as "irrational" or "unreasonable" by members of the health care team.

The family, on the other hand, views the situation very differently. They will tend to focus on the positive, holding out hope that their loved one will beat the odds. If the physician tells them their loved one has a 90% chance of mortality, what they hear is that their loved one has a 10% chance of survival. They aren't guided by success rates or statistics or prognostics; they are guided by devotion and/or a sense of duty to their loved one, a protective instinct, and hope. They may also be guided by deeply held religious beliefs, which they claim are also held by the patient. From the family's perspective, the health care professionals are being insensitive and disrespectful, unwilling to listen to or accept what is important to them. Sometimes families will go so far as to accuse the health care team of wanting to withdraw treatment to save money or to give the resources to another patient. Many of the panel members reported having been involved in ethics consultations where family members have expressed these sentiments.

What lies at the root of these conflicts is a clash of value systems. It is our value systems that influence the decisions we make, especially when we are faced with significant life-altering decisions in the health care setting. But it is not just patients and their families that are guided in their decisions by their values; health care professionals also come to their encounters with patients and families with their own value systems, both personal and professional. The fact that Canada is one of the most culturally diverse nations in the world means that clashes between the value systems of patients/families and health care professionals may be more common in Canadian health care institutions than in other countries.

### Addressing the top challenge

Compared to the attention given to many of the challenges listed in the top ten, it is remarkable how little attention has been given to the top challenge. It is especially remarkable given that these conflicts occur in health care institutions across the country on a daily basis. Below, we propose several steps to help address this top challenge.

1. *Educating health care professionals*: Although most health care professionals are now taught communication skills, they are not taught the negotiation and mediation skills needed to address serious disagreements. The key is to make an attempt to understand the patient's perspective. We recommend that all health professional programs – undergraduate, postgraduate and continuing – takes steps to address this deficiency;

2. *Creating policies for health care institutions*: Some institutions have developed policies on cases of disagreement, especially at the end of life, but there is no consistency in this area across institutions. Some national accreditation organizations, such as the Canadian Council on Health Services Accreditation  and the Joint Commission on Accreditation of Healthcare Organizations in the U.S. , require health care institutions to have systems in place to address ethical issues facing patients, family members, and staff. We recommend this requirement be sharpened to include mechanisms to resolve disagreements between the health care team and patents or their substitute decision makers. In addition, it may be worthwhile to explore the plausibility of approaching policy development in this area through a process of public consultation. Having stakeholders with diverse value systems come together to discuss the challenge may prove to be a more fruitful approach than applying the standard top-down approach;

3. *Examining the patient's perspective*: Disagreements between patients or their substitute decision makers and health care teams present a difficult problem with no perfect solution. What is needed is a better understanding of the patient's perspective on this challenge. Some excellent work has been done in the attempt to shift the focus of end-of-life issues from the perspective of health care professionals and bioethicists to patients themselves [9,10]. However, what is still needed is quality research that focuses specifically on the perspectives of patients toward disagreements over treatment decisions;

4. *Reporting to the public*: Research studies like ours are only one part of a strategy to address the top challenge. A key part of the strategy would be a systematic effort to keep the public informed of such research and the attempts being made to address the challenge. National health councils or other similar bodies would be an excellent mechanism to pull together the diverse initiatives described above and to keep the public informed. Not only should the public be kept informed of steps to address the challenge but they should ideally be involved in the process itself. As mentioned above, one example of involving the public in the process would be to engage the public in the development of policies or guidelines to help address the top challenge.

### Limitations of the study

The main limitations of this study relate to the generalizability of the results. First, the ranking of challenges may not be generalizable to contexts outside of Canada. Some of the challenges listed may be challenges that are particular to the Canadian context because of our Medicare system, such as the challenge of waiting lists or the shortage of family physicians. If this same study were conducted in other countries, it is possible that these challenges would be ranked much lower than in our study, or may not be ranked as a major challenge at all. However, we believe that on the whole our results are likely generalizable at least to other industrialized nations. The challenge of medical error, for example, is a universal challenge because medicine is, by its nature, a human endeavour. As long as humans remain imperfect, medical errors will occur. Moreover, we would predict that the top ranked challenge, disagreements between patients/families and health care providers over treatment decisions, would probably appear at or near the top of similar lists in other industrialized nations. Thus, although the panel was asked to report on the top ethical challenges facing Canadians in health care, we believe the results of this study would be of interest to other countries.

Second, because the panel was made up of clinical bioethicists in Toronto, the ranking of challenges may not be representative of the challenges facing the entire Canadian public. Some of the challenges might be considered more or less significant or prevalent in other parts of Canada, especially since there are some very apparent differences between the health care systems of the different provinces. Nevertheless, for the same reasons as mentioned above in the context of generalizability to other nations, we believe the results are in general representative of the challenges facing the Canadian public.

Third, since our panel was made up entirely of clinical bioethicists, we recognize that the results may not be generalizable to other groups. For example, if the panel was comprised of, or included, members of the public, hospital administrators, or clinicians, the results might have looked very different. Moreover, although the panel members do represent a wide range of health care instutitions, there were health care settings not represented amongst the group (e.g., home care or community family medicine). However, we believe that this is not a significant limitation of the study because the purpose was not to make a factual claim about what, objectively speaking, are the top ten ethical challenges facing the public. Rather, the purpose was to identify what those top ten challenges are from the perspective of a group of highly qualified and experienced clinical bioethicists who work in a variety of health care institutions.

Finally, we recognize that the modified Delphi process that we have presented in this paper is not typical because of the face-to-face meeting of panel members that took place prior to the final round of ranking. One of the potential limitations of including a face-to-face meeting during a consensus process is that a member or members of the group could exert influence over others, thus skewing the process away from genuine consensus. Nevertheless, we believe this potential problem was mitigated by the fact that the face-to-face meeting was not actually part of the ranking process but was an intermediate step between ranking rounds for the purpose of clarifying and differentiating the items. Thus, the consensus process itself was not directly affected by the face-to-face meeting.

## Summary

Patients and their families face a number of ethical challenges in health care. Many of these challenges are no different from the kinds of challenges faced by patients and families in other industrialized nations. Other challenges on the list are more particular to our social context, with their roots in the very nature of the Canadian Medicare system. Waiting lists, access to needed care for the aged, chronically ill, and mentally ill, and the shortage of family physicians, are challenges that may impact Canadians to a greater or lesser degree than citizens of other nations. Interestingly, these three context-specific challenges were all ranked in the top four of the top ten ethical challenges facing Canadians. Moreover, some of the challenges have received far more public attention than others. Since so little attention has been given to the top ranked challenge, disagreements between patients/families and health care professionals over treatment decisions, we have suggested several steps to help address this top challenge.

## Competing interests

The author(s) declare that they have no competing interests.

## Authors' contributions

JMB contributed substantially to the drafting of the article and gave final approval for the version to be published.

SKM contributed substantially to the conception and design, analysis and interpretation of data, drafting of the article and revising it critically and gave final approval for the version to be published.

JB contributed substantially to the conception and design, analysis and interpretation of data, and gave final approval for the version to be published.

PAS contributed substantially to the conception and design, analysis and interpretation of data, drafting of the article and revising it critically and gave final approval for the version to be published.

## Pre-publication history

The pre-publication history for this paper can be accessed here:


